# Positive Interaction between H_2_O_2_ and Ca^2+^ Mediates Melatonin-Induced CBF Pathway and Cold Tolerance in Watermelon (*Citrullus lanatus* L.)

**DOI:** 10.3390/antiox10091457

**Published:** 2021-09-14

**Authors:** Jingjing Chang, Yanliang Guo, Jiayue Li, Zhuangzhuang Su, Chunxia Wang, Ruimin Zhang, Chunhua Wei, Jianxiang Ma, Xian Zhang, Hao Li

**Affiliations:** 1 State Key Laboratory of Crop Stress Biology for Arid Areas, College of Horticulture, Northwest A&F University, Xianyang 712100, China; changjing0710@nwsuaf.edu.cn (J.C.); yanliangg@nwafu.edu.cn (Y.G.); lijiayue@nwafu.edu.cn (J.L.); suz@nwafu.edu.cn (Z.S.); chunxiawang@nwafu.edu.cn (C.W.); zrm0923@nwafu.edu.cn (R.Z.); xjwend020405@nwafu.edu.cn (C.W.); majianxiang@nwsuaf.edu.cn (J.M.); 2 State Key Laboratory of Vegetable Germplasm Innovation, Tianjin 300384, China

**Keywords:** melatonin, hydrogen peroxide, calcium signal, CBF-responsive pathway, respiratory burst oxidase homolog D, cold stress

## Abstract

Cold stress is a major environmental factor that detrimentally affects plant growth and development. Melatonin has been shown to confer plant tolerance to cold stress through activating the C-REPEAT BINDING FACTOR (CBF) pathway; however, the underlying modes that enable this function remain obscure. In this study, we investigated the role of H_2_O_2_ and Ca^2+^ signaling in the melatonin-induced CBF pathway and cold tolerance in watermelon (*Citrullus lanatus* L.) through pharmacological, physiological, and genetic approaches. According to the results, melatonin induced H_2_O_2_ accumulation, which was associated with the upregulation of *respiratory burst oxidase homolog D* (*ClRBOHD*) during the early response to cold stress in watermelon. Besides, melatonin and H_2_O_2_ induced the accumulation of cytoplasmic free Ca^2+^ ([Ca^2+^]_cyt_) in response to cold. This was associated with the upregulation of *cyclic nucleotide-gated ion channel 2* (*ClCNGC2*) in watermelon. However, blocking of Ca^2+^ influx channels abolished melatonin- or H_2_O_2_-induced CBF pathway and cold tolerance. Ca^2+^ also induced *ClRBOHD* expression and H_2_O_2_ accumulation in early response to cold stress in watermelon. Inhibition of H_2_O_2_ production in watermelon by RBOH inhibitor or in Arabidopsis by *AtRBOHD* knockout compromised melatonin-induced [Ca^2+^]_cyt_ accumulation and melatonin- or Ca^2+^-induced CBF pathway and cold tolerance. Overall, these findings indicate that melatonin induces *RBOHD*-dependent H_2_O_2_ generation in early response to cold stress. Increased H_2_O_2_ promotes [Ca^2+^]_cyt_ accumulation, which in turn induces H_2_O_2_ accumulation via *RBOHD*, forming a reciprocal positive-regulatory loop that mediates melatonin-induced CBF pathway and subsequent cold tolerance.

## 1. Introduction

Plants are sessile organisms and, therefore, must withstand multiple environmental stresses throughout their life cycle. As a major environmental constraint, cold stress causes adverse effects on plant growth and development, threatening agricultural production worldwide [1]. Based on temperature and various physiological mechanisms, cold stress in plants is classified as chilling stress (temperatures below optimum but above 0 °C) and freezing stress (<0 °C) [2]. Watermelon (*Citrullus lanatus* L.) is an economically important crop globally. Its origin can be traced to tropical and subtropical regions of Africa. Watermelon production is threatened by its susceptibility to low temperatures [3]. Chilling temperatures adversely affect watermelon seedlings, including reduction of photosynthetic ability, oxidative damage, membrane dysfunction, and hormonal imbalance, leading to reduced growth and development, delay in flowering and fruit set, and decrease in fruit quality and total output [4,5,6].

Plants have evolved sophisticated defense mechanisms for surviving cold stress. They can perceive temperature declines, leading to signal transduction from the plasma membrane to the nucleus, activating diverse transcriptional regulators, and inducing osmotic factors [2]. Calcium (Ca^2+^) signaling plays an essential function in signal transduction in plant response to cold stress. Temperature decrease activates Ca^2+^ channels in plants, leading to rapid induction of Ca^2+^ signals, which confers cold tolerance by activating downstream phosphorylation cascade and transcriptional regulation pathways, such as the C-repeat binding factors (CBFs)/Drought response element-binding factors (DREBs)-dependent signaling pathway [7,8]. CBFs/DREBs control the expression of numerous *cold-responsive* (*COR*) genes by specifically recognizing and binding to the C-repeat/dehydration responsive element (DRE) motif [9]. Overexpression of *CBFs* enhanced plant tolerance to cold stress, while mutation of *CBFs* reduced cold tolerance [10,11]. Furthermore, the CBF-response pathway is highly conserved among plants [12].

Besides Ca^2+^ influx, oxidative burst is one of the initial plant responses to cold exposure. It involves enhanced production and steady state levels of reactive oxygen species (ROS), such as superoxide (O_2_^−^), hydrogen peroxide (H_2_O_2_), and hydroxyl radicals (OH·). H_2_O_2_ produced by the NADPH oxidase encoded by *respiratory burst oxidase homologues* (*RBOHs*), is an important signaling molecule that triggers downstream responses such as CBF-dependent pathway to confer cold tolerance [13,14]. Plant RBOHs have a highly conserved cytosolic N-terminal with two Ca^2+^-binding EF-hand motifs [15]. Cold-induced Ca^2+^ activates RBOH activity by direct binding to the N-terminal EF-hands to trigger H_2_O_2_ generation, which in turn elicits Ca^2+^ transients in plant cells [8,16,17].

In the last two decades, melatonin (*N*-acetyl-5-methoxytryptamine) has emerged as an essential bio-stimulant in plants because of its essential regulatory functions in growth, development, and response to various environmental agents [18]. The recent identification of the first phytomelatonin receptor (CAND2/PMTR1) in *Arabidopsis* validated phytomelatonin as a new plant hormone [19,20]. Several lines of evidence prove that melatonin has a protective function against cold stress in plants. Exogenous melatonin has been shown to strengthen chilling tolerance in diverse plant species by scavenging excessive ROS and improving the photosynthesis system [21,22,23]. A recent study has shown that melatonin, as a potential root-to-shoot signal, induces the increases in methy jasmonate and H_2_O_2_ in rootstock-scion communication in response to cold stress [24]. Melatonin induces the expression of *CBFs* and *COR* genes under cold stress, suggesting that the CBF pathway may participate in melatonin-induced cold tolerance [25,26,27]. Furthermore, the upregulation of *COR* genes induced by melatonin is in an ABA-independent manner [28]. However, the mechanism by which melatonin activates the CBF pathway and subsequently enhances plant tolerance to cold stress remains unclear.

Increasing evidence indicates that H_2_O_2_ and Ca^2+^ signaling facilitate melatonin-mediated regulation of multiple physiological processes, including seed germination and stomatal closure [19,29]. These findings raise a presumption that H_2_O_2_ and Ca^2+^ signaling may also have a critical role in the melatonin-induced CBF-responsive pathway and subsequent chilling tolerance. To verify this presumption, we examined the functions of H_2_O_2_ and Ca^2+^ and their interaction in melatonin-induced chilling tolerance. Our results reveal that the interaction between H_2_O_2_ and Ca^2+^ mediates the melatonin-induced CBF pathway and subsequent cold tolerance. This study provides new insights into understanding the regulation mechanism of melatonin-induced cold tolerance.

## 2. Materials and Methods

### 2.1. Plant Materials and Growth Conditions

Watermelon (*Citrullus lanatus* cv. Nongkeda No. 5) seeds were collected from the Watermelon and Melon Research Group of Northwest A&F University, Yangling, China. Germinated seeds were planted into plastic pots filled with a mixture of peat and vermiculite (3:1). The seedlings were cultivated in a growth chamber under photoperiod of 12/12 h (day/night), temperature of 28/18 °C (day/night), and a photosynthetic photo flux density (PPFD) of 600 μmol m^−2^ s^−1^. *Arabidopsis* seeds of *Atrbohd* (SALK_120299) mutant with Columbia-0 (Col-0) genetic background were received from the Arabidopsis Biological Resource Center (https://www.arabidopsis.org/, accessed date 16 May 2019) [29]. After surface sterilization with 75% ethanol and 3% sodium hypochlorite, *Arabidopsis* seeds were sown on half-strength Murashige-Skoog (MS) medium containing 0.8% agar and 1.0% sucrose and cultured at 22 °C in a growth chamber under 16/8 h photoperiod.

### 2.2. Experimental Treatment

To evaluate the influences of melatonin, H_2_O_2_, or Ca^2+^ on watermelon response to cold stress, the seedlings were sprayed with 150 µM melatonin [24], H_2_O_2_ (0.04, 0.2, 1, 5 mM), 20 mM CaCl_2_ [30,31], or distilled water (as the control). Melatonin (Sigma-Aldrich, St. Louis, MO, USA) was dissolved in ethanol and then diluted with distilled water at a ratio of 1:10,000 [ethanol:water; *v*:*v*]. After 12 h, the watermelon seedlings were exposed to chilling treatment at 4 °C for 48 h. The leaves were sampled at 3, 6, 12, and 24 h time points during chilling exposure to analyze the expression of CBF pathway genes and at 48 h for cold tolerance assay. After the initial experiments, 1 mM H_2_O_2_ was selected for the subsequent experiments.

To determine the role of H_2_O_2_ in melatonin- or Ca^2+^-induced cold tolerance, watermelon seedlings were pretreated with 100 µM diphenyleneiodonium (DPI, an inhibitor of NADPH oxidases, which catalyzes H_2_O_2_ production) [32] 2 h before melatonin or Ca^2+^ application. After 12 h, the seedlings were transferred to 4 °C for 48 h. To investigate the role of Ca^2+^ in melatonin- or H_2_O_2_-induced cold tolerance, the watermelon seedlings were sprayed with 10 mM lanthanum chloride (LaCl_3_, a Ca^2+^ channel blocker) [33] 2 h before melatonin or H_2_O_2_ treatment. After 12 h, the seedlings were transferred to 4 °C for 48 h.

To explore the function of *RBOHD* in melatonin- or Ca^2+^-induced freezing tolerance in *Arabidopsis*, three-week-old wild-type or *Atrbohd* mutant *Arabidopsis* plants were pretreated with 10 μM melatonin [34] or 1 mM CaCl_2_ [35]. After 12 h, the seedlings were subjected to freezing at −10 °C for 1 h and then recovered at 22 °C for 5 days [36]. The proportion of plants with green leaves was recorded to determine the survival rate. To determine the role of *RBOHD* in melatonin- or Ca^2+^-induced CBF pathway in response to cold, the *Arabidopsis* seedlings pretreated with melatonin or CaCl_2_ were exposed to 4 °C for 24 h. Leaves were sampled at 3, 6, 12, and 24 h after 4 °C treatment.

Protoplasts from watermelon or *Arabidopsis* leaves were extracted and used to examine the effects of melatonin or H_2_O_2_ on the accumulation of cytosolic free calcium ([Ca^2+^]_cyt_) in response to chilling stress. The protoplasts were incubated with Fluo-4 acetoxymethyl (AM) ester (a Ca^2+^-sensitive fluorescent dye) at 37 °C in the dark. After 30 min, the protoplasts loaded with Fluo-4/AM were treated with melatonin (10 µM), H_2_O_2_ (100 µM), or a combination of melatonin and DPI (10 µM) and then placed at 4 °C for 5 min.

### 2.3. Cold Tolerance Assay

The maximum photosystem II quantum yield (Fv/Fm) was determined on the upper second fully expanded leaves of watermelon seedlings after 30 min of dark adaptation using a FluorCam fluorescence imaging system (SN-FC800-240; Photon Systems Instruments; Brno, Czech Republic) [37]. The relative electrical conductivity (REC) was determined as described by Zhou and Leul [38]. The level of lipid peroxidation in plant cells was assessed by determining malondialdehyde (MDA) contents using a 2-thiobarbituric acid (TBA) reaction [39].

### 2.4. Hydrogen Peroxide Content Assay

Hydrogen peroxide (H_2_O_2_) content was determined as described by Willekens et al. [40]. In brief, leaf samples (0.5 g each) were ground in 5 mL of ice-cold 1 M HClO_4_. Next, the homogenate was centrifuged at 6000× *g* for 5 min at 4 °C and neutralized to pH 6.0–7.0 with 4 M KOH. After that, the homogenate was further centrifuged at 12,000× *g* for 5 min at 4 °C, and the supernatant was loaded on an AG1-X8 prepacked column (Bio-Red, Hercules, CA, USA) and eluted with 4 mL double-distilled water. The sample extract (800 µL) was added to the reaction mixture of 400 µL 100 mM potassium acetate buffer (pH 4.4) containing 4 mM 2,2′-azino-di (3-ethylbenzthiazoline-6-sulfonic acid), 400 µL deionized water, and 0.25 U of horseradish peroxidase (HRP). The absorbance at OD_412_ was recorded to calculate H_2_O_2_ content.

### 2.5. Protoplast Isolation and Measurement of [Ca^2+^]_cyt_

Protoplasts were isolated as described previously with modifications [41,42]. Briefly, leaves from three-week-old watermelon or four-week-old *Arabidopsis* seedlings were cut into 0.5 mm wide strips. Watermelon leaf strips were digested with 10 mL enzyme solution containing 1.5% cellulose R10 and 0.3% macerozyme R10, whereas *Arabidopsis* leaf strips were digested with enzyme solution containing 1.0% cellulose R10 and 0.2% macerozyme R10. The digestion was performed for 3–4 h in the dark, after which the enzyme solutions were diluted with 10 mL W5 solution (pH 5.7) containing 2 mM MES, 154 mM NaCl, 125 mM CaCl_2_, and 5 mM KCl, then filtered through a 75 µm nylon mesh. The protoplasts were collected after centrifugation at 100× *g* for 5 min at 4 °C and re-suspended in 10 mL W5 solution.

The [Ca^2+^]_cyt_ in the protoplasts was measured using Fluo-4 AM [43]. The protoplasts were incubated with 4 µM Fluo-4 AM and 0.02% Pluronic F-127 (Shanghai Yeasen Science & Technology Co., Ltd., Shanghai, China) at 37 °C in the dark for 30 min. Fluorescence from the protoplasts loaded with Fluo-4/AM was observed under a confocal microscope (TCS SP8 SR, Leica, German), at excitation of 488 ± 10 nm and emission of 520 ± 10 nm. The fluorescence intensity was calculated using Image J v1.8.0 software (National Institutes of Health, Bethesda, MD, USA) to reflect [Ca^2+^]_cyt_ accumulation levels.

### 2.6. RNA Extraction and qRT-PCR Assay

Total RNA was extracted from watermelon or *Arabidopsis* leaves using an RNA simple Total RNA kit (TIANGEN, Beijing, China). After extraction, the total RNA samples were treated with gDNase, then reverse-transcribed (1 µg per sample) to cDNA using a FastKing RT kit (TIANGEN, Beijing, China). The qRT-PCR assay was performed on a StepOnePlus^TM^ Real-Time PCR System (Applied Biosystems, Carlsbad, CA, USA) using SYBR^®^ Premix ExTaq^TM^ II (2×) kit (Takara, Tokyo, Japan). The gene-specific primers used for the qRT-PCR are listed in Table S1. The qRT-PCR amplification was conducted under the conditions reported by Li et al. [29]. *β-actin* or *AtActin2* served as the internal control genes for the normalization of gene expression [11,44]. Relative gene expression was calculated as described by Livak and Schmittgen [45].

### 2.7. Phylogenetic Analysis

*Arabidopsis* AtRBOH and watermelon ClRBOHD protein sequences were downloaded from Arabidopsis Information Resource (https://www.arabidopsis.org/, accessed date 16 May 2019) and Cucurbit Genomics Database (CuGenDB, http://cucurbitgenomics.org/, accessed date 23 July 2020), respectively. Multiple sequence alignment of these RBOH protein sequences was performed via ClustalW with default parameters [46]. Phylogenetic analysis was conducted using the MEGA7.0.21 software. Based on the result of multiple sequence alignment, a phylogenetic tree was constructed using the Neighbor-Joining method and the parameters were Jones–Taylor–Thornton (JTT) matrix-based model and 1000 bootstraps [47].

### 2.8. Statistical Analysis

The experiments were performed in a completely randomized design. Each experiment was repeated thrice, and each replicate included at least 18 plants. The differences among treatments were determined via one-way ANOVA using SPSS statistics 19 (SPSS Inc., Chicago, IL, USA), followed by Tukey’s test at *p* < 0.05.

## 3. Results

### 3.1. The Requirement of H_2_O_2_ for Melatonin-Induced CBF-Responsive Pathway and Chilling Tolerance in Watermelon

**Figure 1:**
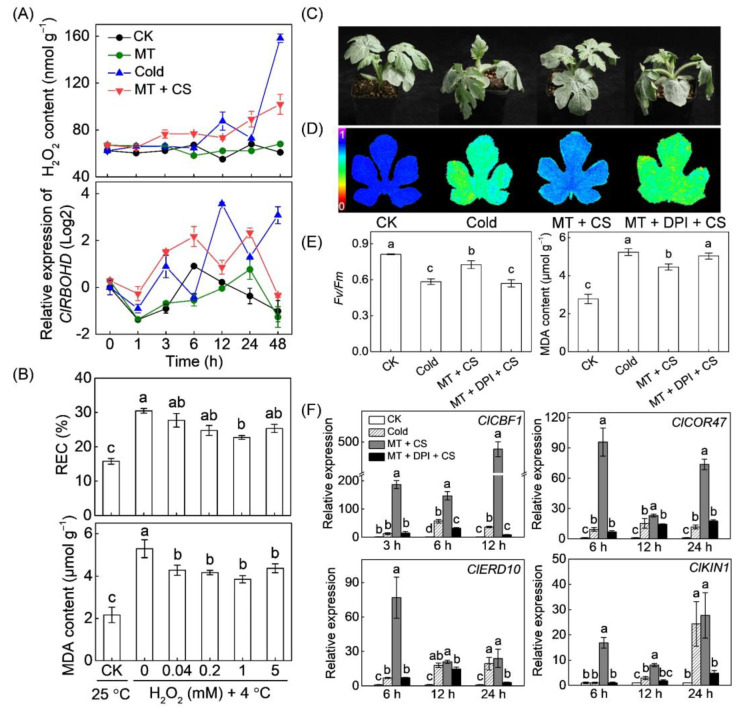
Involvement of H_2_O_2_ in melatonin-induced C-REPEAT BINDING FACTOR (CBF) transcriptional regulatory cascade and chilling tolerance. (**A**) Dynamic changes in H_2_O_2_ accumulation and the expression of *respiratory burst oxidase homolog D* (*ClRBOHD*). Watermelon seedlings at three-leaf stage were sprayed with 150 µM melatonin (MT), and after 12 h, they were subjected to cold stress (CS) at 4 °C for 48 h. Seedlings sprayed with distilled water under temperatures of 28/18 °C (day/night) were set as control (CK). (**B**) The relative electrical conductivity (REC) and malondialdehyde (MDA) content. The seedlings were sprayed with 0.04, 0.2, 1, or 5 mM H_2_O_2_, and after 12 h, they were exposed to 4 °C for 48 h. (**C**) Chilling phenotypes. (**D**) Images showing the maximum PSII quantum yield (Fv/Fm). The false-color code depicted at the left of the image ranges from 0 (black) to 1 (purple). (**E**) The average values of Fv/Fm and MDA contents. (**F**) The expression of *ClCBF1* and its regulons, including *cold-responsive gene 47* (*ClCOR47*), *early responsive to dehydration 10* (*ClERD10*), and *cold induced gene 1* (*ClKIN1*). For (**C**–**F**), the seedlings were sprayed with 100 µM diphenyleneiodonium (DPI, an inhibitor of H_2_O_2_ production) 2 h before MT treatment. After 12 h, the seedlings were subjected to 4 °C. Relative expression of genes in CK plants was set as 1.0. Data show the means of three replicates ± standard deviation (SD). The different letters denote significant difference at *p* < 0.05 according to Turkey’s test.

The effect of melatonin on H_2_O_2_ accumulation was first examined to determine whether H_2_O_2_ is necessary for melatonin function in watermelon response to chilling stress. The results showed no significant differences in H_2_O_2_ accumulation between melatonin-pretreated and control plants under optimum growth conditions (Figure 1A). Chilling exposure induced an H_2_O_2_ burst from 6 h; however, such induction was accelerated by melatonin. Pretreatment with melatonin triggered an H_2_O_2_ burst from 1 h after chilling treatment. For instance, after chilling exposure at 3 and 6 h, H_2_O_2_ contents in melatonin-pretreated plants increased by 15.9% and 19.0%, respectively, compared to the control plants. However, H_2_O_2_ contents in melatonin-pretreated plants were less than that in the control plants at 12 and 48 h after chilling treatment. The changes in *ClRBOHD* transcript levels showed similar trends with H_2_O_2_ in response to melatonin or/and chilling stress. For example, the transcript levels of *ClRBOHD* in melatonin-pretreated plants were 0.5 and 5.2 fold higher than that in the control plants at 3 and 6 h, respectively, after chilling exposure.

Application of H_2_O_2_ at optimum concentrations (0.04–5 mM) alleviated chilling-induced increases in REC and MDA. The most effective H_2_O_2_ concentration was 1 mM (Figure 1B). Notably, H_2_O_2_ concentrations higher or lower than 1 mM attenuated the positive effect of H_2_O_2_ on chilling tolerance. Similarly, melatonin application enhanced watermelon defense against chilling stress, as exhibited by the alleviation of leaf wilting, an increase in Fv/Fm, and a decrease in MDA content (Figure 1C–E). However, pretreatment with DPI (an inhibitor of H_2_O_2_ production, 100 μM) completely abolished the melatonin-induced chilling tolerance. The CBF-responsive pathway plays a central role in plant defense against cold stress [9]. Chilling stress induced the expression of *ClCBF1* and its regulons, including *cold-responsive gene 47* (*COR47*), *early responsive to dehydration 10* (*ERD10*), and *cold induced gene 1* (*KIN1*). Importantly, transcript levels of the genes mentioned above were significantly higher in the melatonin-pretreated plants than in the control plants after chilling treatment. However, pretreatment with DPI prevented the melatonin-induced increases in the transcripts of CBF-responsive pathway genes.

### 3.2. Involvement of Ca^2+^ Signal in Melatonin- and H_2_O_2_-Induced Chilling Tolerance in Watermelon

**Figure 2:**
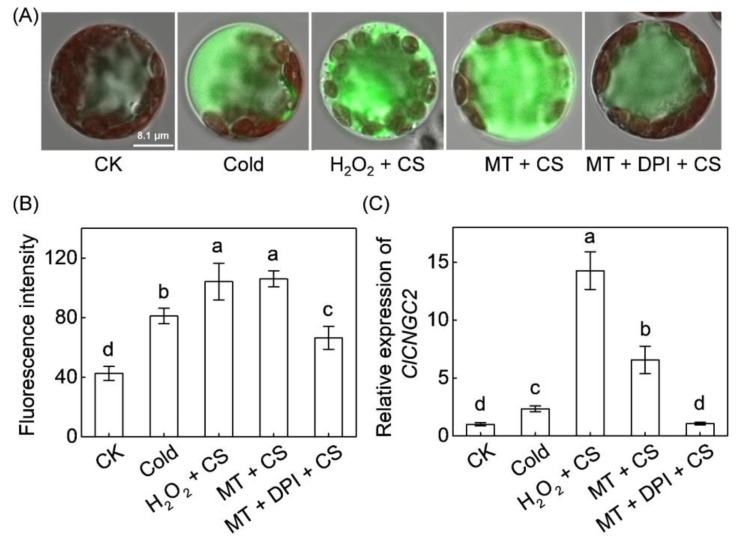
The involvement of H_2_O_2_ in melatonin-induced accumulation of cytoplasmic free Ca^2+^ ([Ca^2+^]_cyt_) in response to chilling stress. (**A**) [Ca^2+^]_cyt_ accumulation in protoplasts from watermelon leaves. (**B**) Fluorescence intensity. The protoplasts were incubated with Fluo-4 acetoxymethyl (AM) ester at 37 °C in the dark. After 30 min, the protoplasts were treated with melatonin (10 µM), H_2_O_2_ (100 µM), or a combination of melatonin and DPI (10 µM) and then placed at 4 °C for 5 min (cold stress, CS). Data in (**B**) are reported as means ± standard deviation (SD, *n* ≥ 7). (**C**) The relative expression of *cyclic nucleotide-gated channel 2* (*ClCNGC2*) at 6 h after chilling exposure. The seedling leaves were sprayed with 150 µM melatonin (MT) or 1 mM H_2_O_2_. After 12 h, the seedlings were transferred to 4 °C. To inhibit H_2_O_2_ production, the seedlings were sprayed with DPI at 100 µM 2 h before melatonin application. Data are reported as means ± standard deviation (SD, *n* = 3). The different letters denote significant difference at *p* < 0.05 according to Turkey’s test. CK, control.

To investigate whether Ca^2+^ signal is involved in melatonin- or H_2_O_2_-mediated cold tolerance, the [Ca^2+^]_cyt_ accumulation in watermelon protoplasts was first measured using the Fluo-4 AM ester as a fluorescent indicator of Ca^2+^. According to the results, chilling exposure induced the accumulation of [Ca^2+^]_cyt_ in watermelon protoplasts (Figure 2). Notably, both melatonin and H_2_O_2_ pretreatment significantly increased the cold-induced accumulation of [Ca^2+^]_cyt_. For example, the fluorescence intensity of [Ca^2+^]_cyt_ in protoplasts pretreated with melatonin and H_2_O_2_ increased by 46% and 32%, respectively, compared with the control protoplasts after chilling stress. However, DPI application prevented the melatonin-induced [Ca^2+^]_cyt_ accumulation under chilling stress. *Cyclic nucleotide-gated channel 2* (*CNGC2*) encodes a plasma membrane cation channel that directs extracellular Ca^2+^ into the cytosol [48,49]. In line with [Ca^2+^]_cyt_ accumulation, both melatonin and H_2_O_2_ promoted the cold-induced *ClCNGC2* upregulation; however, the effect was blocked by DPI pretreatment. These results demonstrate that H_2_O_2_ participates in melatonin-induced [Ca^2+^]_cyt_ accumulation under chilling stress.

**Figure 3:**
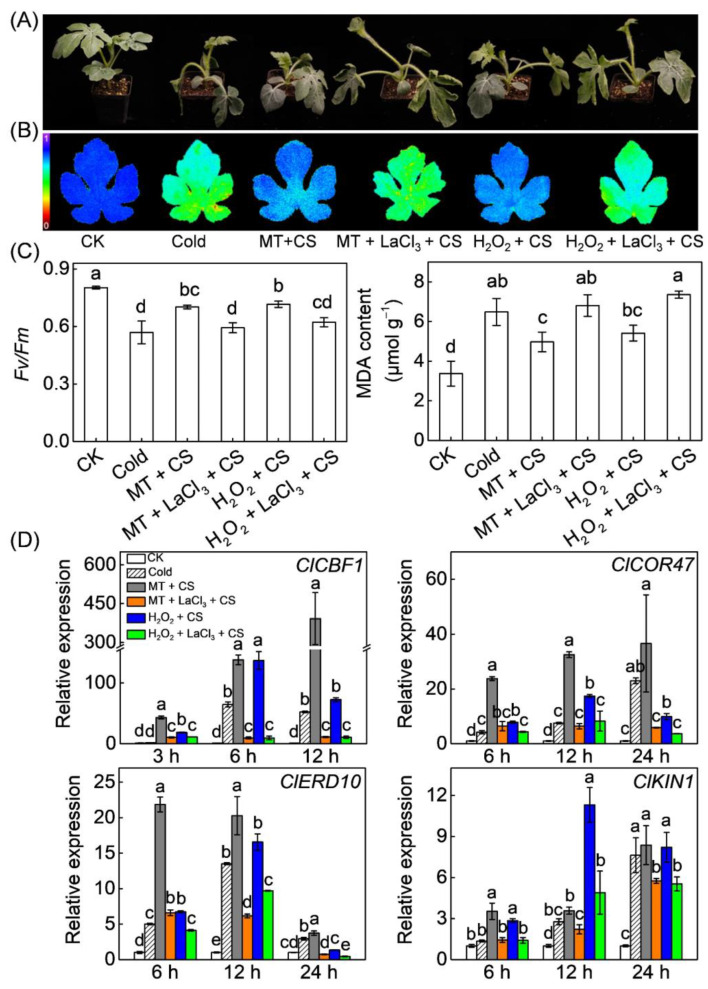
The role of Ca^2+^ in melatonin- or H_2_O_2_-induced C-REPEAT BINDING FACTOR (CBF) pathway and chilling tolerance in watermelon. (**A**) Chilling phenotypes. (**B**) Images showing the maximum PSII quantum yield (Fv/Fm). The false-color code depicted at the left of the image ranges from 0 (black) to 1 (purple). (**C**) The average values of Fv/Fm and malondialdehyde (MDA) contents. (**D**) The expression of *ClCBF1* and its regulons, including *cold-responsive gene 47* (*ClCOR47*), *early responsive to dehydration 10* (*ClERD10*), and *cold induced gene 1* (*ClKIN1*). The plants were treated with melatonin (MT, 150 µM) or H_2_O_2_ (1 mM) for 12 h and then transferred to cold stress (CS) at 4 °C. To block Ca^2+^ influx, the plants were sprayed with 10 mM lanthanum chloride (LaCl_3_) 2 h before MT or H_2_O_2_ application. Seedlings sprayed with distilled water under temperatures of 28/18 °C (day/night) were set as control (CK). Relative expression of genes in CK plants was set as 1.0. Data show the means of three replicates ± standard deviation (SD). The different letters denote significant difference at *p* < 0.05 according to Turkey’s test.

LaCl_3_ is an inhibitor of Ca^2+^ influx channels [50]. In this study, pretreatment with LaCl_3_ completely abolished melatonin- and H_2_O_2_-induced chilling tolerance, characterized by increased leaf wilting, reduced Fv/Fm, and high MDA content (Figure 3A–C). Moreover, application of H_2_O_2_ significantly upregulated the expression of *ClCBF1* and its regulons, including *ClCOR47*, *ClERD10*, and *ClKIN1* after chilling exposure (Figure 3D). Specifically, the expression of *ClCBF1*, *ClCOR47*, *ClERD10*, and *ClKIN1* were upregulated 137.1, 7.8, 6.7, and 2.9 fold, respectively, in H_2_O_2_-pretreated plants, far higher than that (64.6, 4.2, 5.0, and 1.3 fold, respectively) in control plants, at 6 h after chilling exposure. However, LaCl_3_ pretreatment prevented the melatonin- or H_2_O_2_-induced upregulation of *ClCBF1* and its regulons.

### 3.3. Role of H_2_O_2_ in Ca^2+^ Signal-Induced Chilling Tolerance in Watermelon

**Figure 4:**
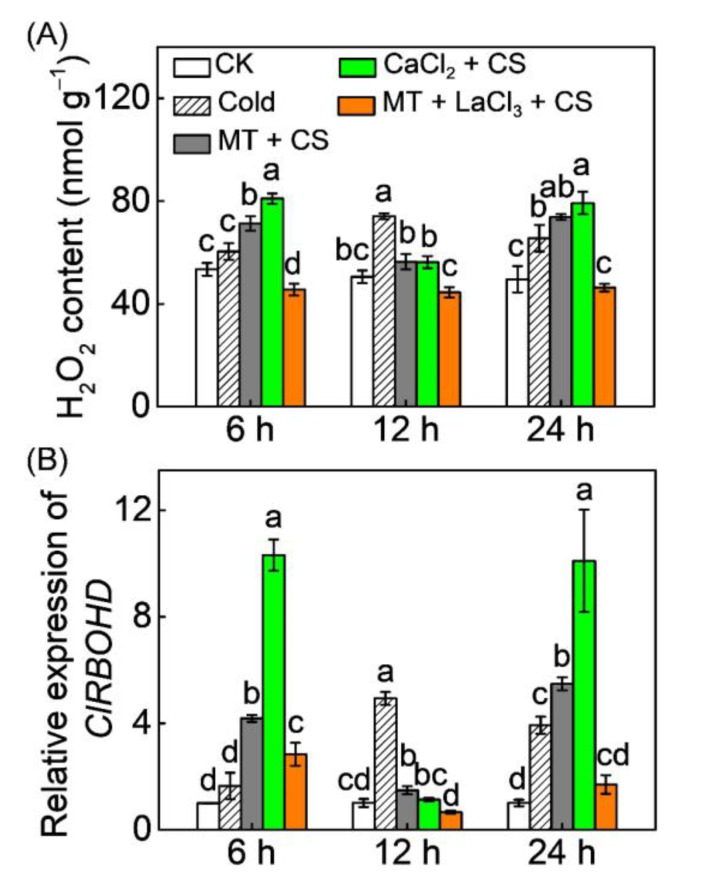
Ca^2+^ mediates melatonin-induced (**A**) H_2_O_2_ accumulation and (**B**) expression of *respiratory burst oxidase homolog D* (*ClRBOHD*) in watermelon leaves under chilling stress. The leaves of watermelon seedlings were sprayed with 150 µM melatonin (MT) or 20 mM CaCl_2_ for 12 h, and then were subjected to cold stress (CS) at 4 °C. To block Ca^2+^ influx, the leaves were pretreated with LaCl_3_ at 10 mM 2 h before melatonin application. Seedlings sprayed with distilled water under temperatures of 28/18 °C (day/night) were set as control (CK). Data show the means of three replicates ± standard deviation (SD). The different letters denote significant difference at *p* < 0.05 according to Turkey’s test.

To investigate the function of Ca^2+^ in melatonin-induced H_2_O_2_ accumulation in response to chilling stress, the effects of CaCl_2_ and LaCl_3_ on H_2_O_2_ accumulation and *ClRBOHD* expression were analyzed. Like melatonin, CaCl_2_ application accelerated the cold-induced upregulation of *ClRBOHD* and subsequent H_2_O_2_ burst (Figure 4). After chilling exposure, the levels of H_2_O_2_ and *ClRBOHD* transcripts in CaCl_2_-pretreated plants increased by 0.5 and 10.3 fold, respectively, at 6 h, while those in control plants were induced at 12 h. However, LaCl_3_ pretreatment completely blocked melatonin-induced H_2_O_2_ accumulation and *ClRBOHD* upregulation under chilling stress, suggesting that Ca^2+^ signal mediates melatonin-induced *ClRBOHD* expression and H_2_O_2_ accumulation in response to chilling stress.

**Figure 5:**
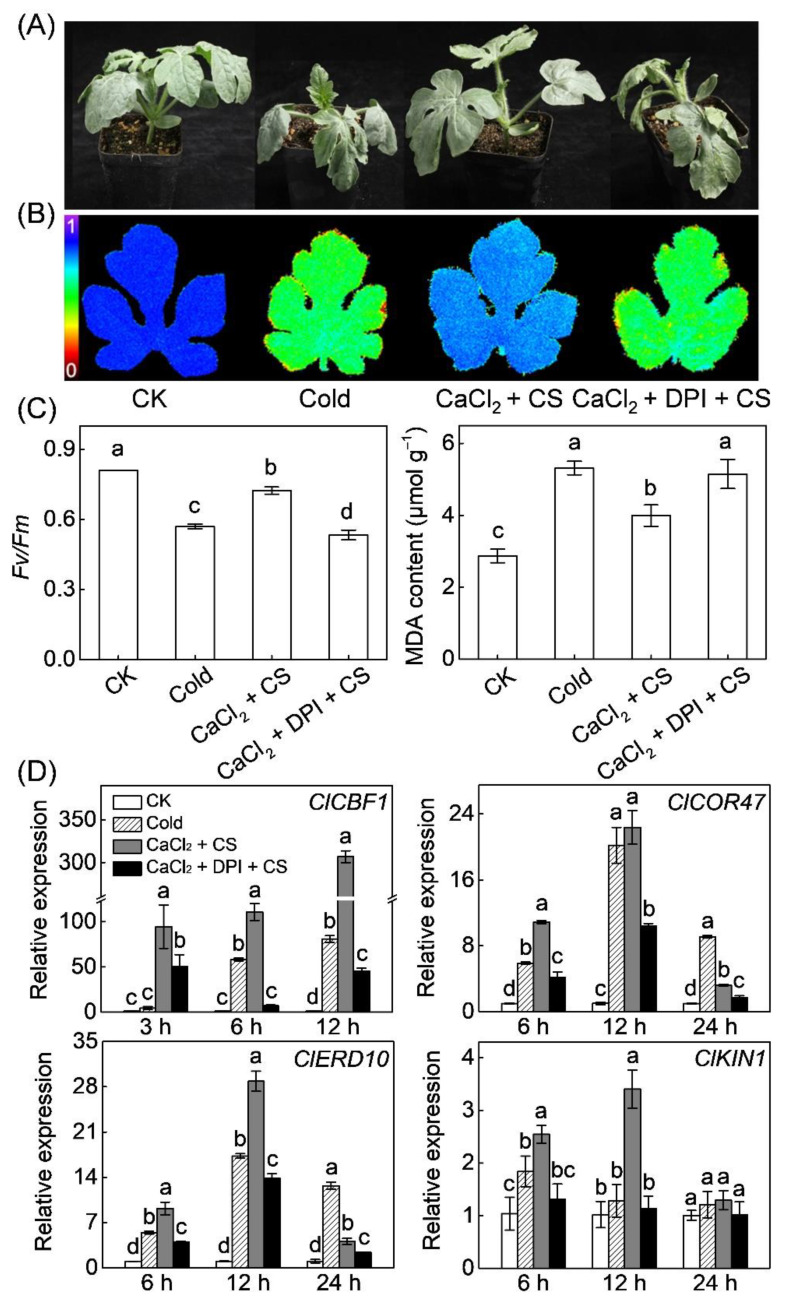
The role of H_2_O_2_ in CaCl_2_-induced C-REPEAT BINDING FACTOR (CBF) pathway and chilling tolerance in watermelon. (**A**) Chilling phenotypes. (**B**) Images showing the maximum PSII quantum yield (Fv/Fm). The false-color code depicted at the left of the image ranges from 0 (black) to 1 (purple). (**C**) The average values of Fv/Fm and malondialdehyde (MDA) contents. (**D**) The expression of *ClCBF1* and its regulons, including *cold-responsive gene 47* (*ClCOR47*), *early responsive to dehydration 10* (*ClERD10*), and *cold induced gene 1* (*ClKIN1*). The plant leaves were sprayed with 20 mM CaCl_2_ for 12 h and then exposed to cold stress (CS) at 4 °C. To inhibit H_2_O_2_ production, the seedlings were pretreated with 100 µM DPI 2 h before CaCl_2_ application. Seedlings sprayed with distilled water under temperatures of 28/18 °C (day/night) were set as control (CK). Relative expression of genes in CK plants was set as 1.0. Data show the means of three replicates ± standard deviation (SD). The different letters denote significant difference at *p* < 0.05 according to Turkey’s test.

Like melatonin and H_2_O_2_, CaCl_2_ alleviated cold-induced leaf wilting, decreased Fv/Fm, and increased MDA content; however, these effects were abolished by DPI treatment (Figure 5A–C). Moreover, CaCl_2_ promoted cold-induced upregulation of CBF pathway genes, including *ClCBF1*, *ClCOR47*, *ClERD10*, and *ClKIN1*, but these effects were blocked by DPI treatment (Figure 5D). These findings suggest that H_2_O_2_ plays a vital function in Ca^2+^ signal-induced CBF-responsive pathway and subsequent chilling tolerance.

### 3.4. Involvement of RBOHD in Melatonin-Induced [Ca^2+^]_cyt_ Accumulation and Freezing Tolerance in Arabidopsis

**Figure 6:**
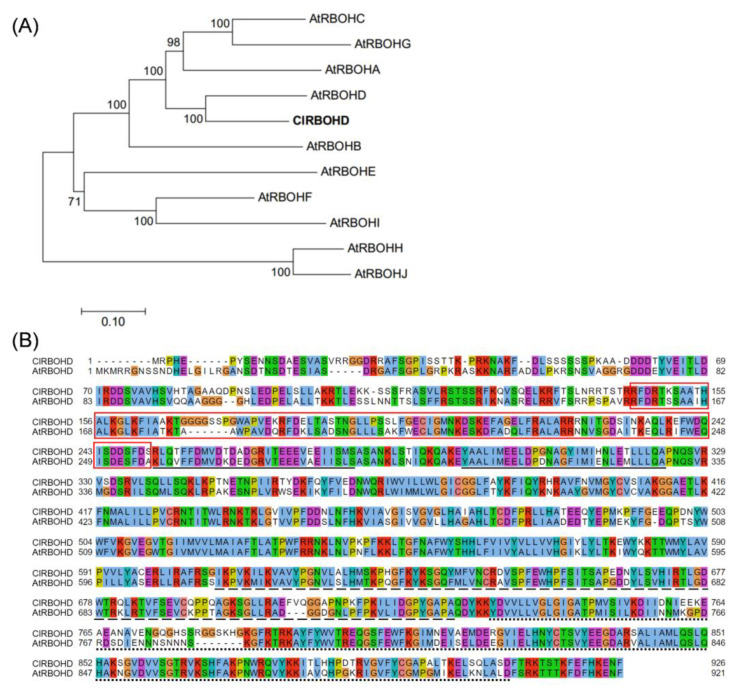
(**A**) Phylogenetic analysis of ClRBOHD and 10 AtRBOH proteins and (**B**) protein sequence alignment and domain structure of ClRBOHD and AtRBOHD. In (**B**), the NADPH_Ox (PF08414) domain is shown in the red box. The EF-hand (IPR002048), FAD_binding_8 (PF08022), and NAD_binding_6 (PF08030) domains are underlined with solid, dashed, and dotted lines, respectively. RBOH, respiratory burst oxidase homolog; Cl, *Citrullus lanatus*; At, *Arabidopsis thaliana*.

Phylogenetic analysis showed that watermelon ClRBOHD has high homology to *Arabidopsis* AtRBOHD with 79.3% similarity (Figure 6A). ClRBOHD and AtRBOHD contain the same critical conserved domains, including NADPH_Ox domain (PF08414), EFh (IPR002048), FAD_binding_8 (PF08022), and NAD_binding_6 (PF08030) (Figure 6B). The NADPH_Ox domain, which is found in respiratory burst NADPH oxidase proteins, shares 75.5% similarity to AtRBOHD and ClRBOHD. Thus, the *Arabidopsis* loss-of-function mutant *Atrbohd* was used to study the role of *RBOHD* in melatonin-induced [Ca^2+^]_cyt_ accumulation and cold tolerance.

**Figure 7:**
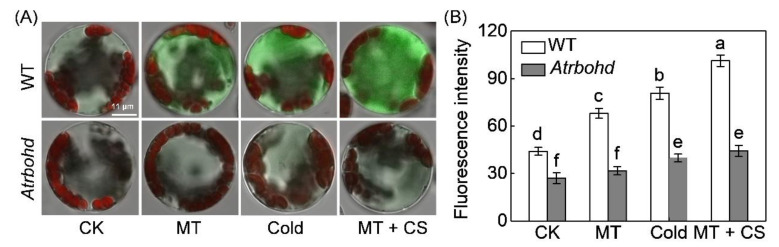
*Respiratory burst oxidase homolog D* (*AtRBOHD*) mediates melatonin-induced accumulation of cytoplasmic free Ca^2+^ ([Ca^2+^]_cyt_) in *Arabidopsis* response to cold stress. (**A**) Fluorescence images. (**B**) Fluorescence intensity. The protoplasts from leaves of wild-type (WT) *Arabidopsis* or *Atrbohd* mutant were incubated with Fluo-4 acetoxymethyl (AM) ester at 37 °C in the dark. After 30 min, the protoplasts were treated with melatonin (10 µM) and then placed at 4 °C for 5 min (cold stress, CS). Data in (**B**) are expressed as means ± standard deviation (SD, *n* ≥ 7). The different letters mean significant difference at *p* < 0.05 according to Turkey’s test. CK, control.

**Figure 8:**
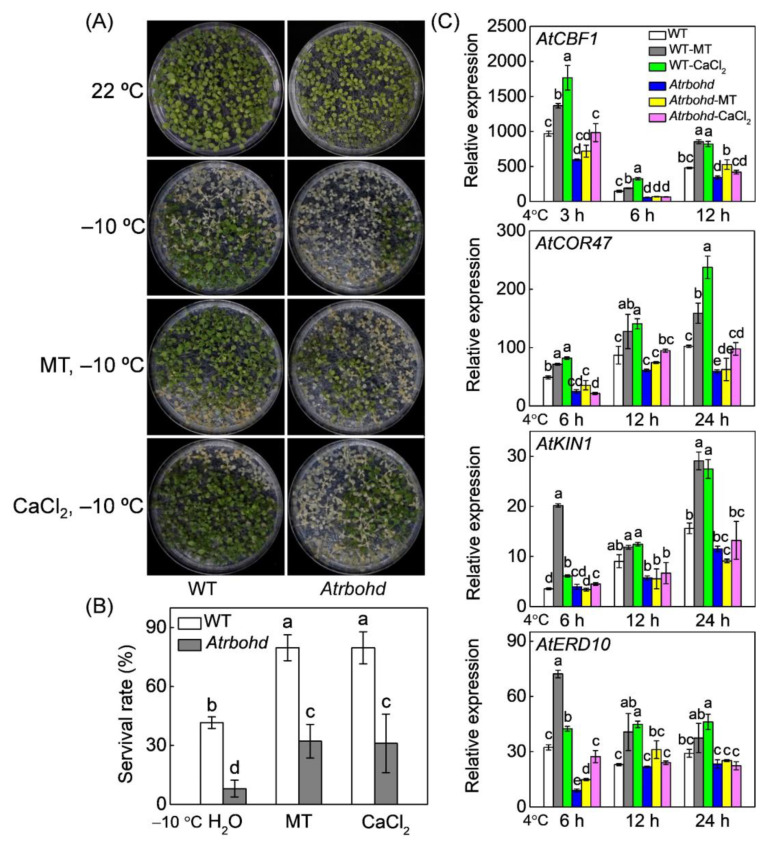
*Respiratory burst oxidase homolog D* (*AtRBOHD*) mediates melatonin- or Ca^2+^-induce freezing tolerance in *Arabidopsis*. (**A**) Freezing phenotypes. (**B**) Survival rates. (**C**) The relative expression of *C-repeat binding factor 1* (*AtCBF1*) and its regulons, including *cold-responsive gene 47* (*AtCOR47*), *early responsive to dehydration 10* (*AtERD10*), and *cold induced gene 1* (*AtKIN1*) under cold stress at 4 °C. Three-week-old wild-type (WT) and *Atrbohd* mutant seedlings grown on the ½ MS medium were treated with 10 µM melatonin (MT) or 1 mM CaCl_2_ (Ca^2+^). After 12 h, the seedlings were subjected to freezing at −10 °C for 1 h and then recovered at 22 °C for 5 days or exposed to chilling at 4 °C for 24 h. Relative expression of genes in untreated WT plants was set as 1.0. Data show the means of three replicates ± standard deviation (SD). The different letters denote significant difference at *p* < 0.05 according to Turkey’s test.

*Atrbohd* mutant protoplasts showed 38.6% and 50.8% less accumulation of [Ca^2+^]_cyt_ than wild-type (WT) protoplasts under normal and chilling conditions, respectively (Figure 7). Similar to watermelon protoplasts, melatonin significantly induced [Ca^2+^]_cyt_ accumulation in WT protoplasts under normal and chilling conditions. However, these effects were abolished by *AtRBOHD* mutation. Moreover, *AtRBOHD* knockout significantly increased *Arabidopsis* sensitivity to freezing at −10 °C and downregulated the expression of *AtCBF1* and its targets, including *AtCOR47*, *AtERD10*, and *AtKIN1* under chilling stress of 4 °C (Figure 8). After freezing exposure, the survival rate of *Atrbohd* mutant was 80.7% lower than the WT plants, suggesting that *AtRBOHD* plays an essential role in *Arabidopsis* response to freezing. Both melatonin and Ca^2+^ application dramatically induced the freezing tolerance and enhanced the expression of CBF pathway genes in WT *Arabidopsis*; however, such effects were attenuated in *Atrbohd* mutants. Overall, these results indicate *AtRBOHD* mediates melatonin-induced [Ca^2+^]_cyt_ accumulation and melatonin/Ca^2+^-induced freezing tolerance.

## 4. Discussion

The CBF transcriptional regulatory cascade plays a central role in the regulation of cold stress response in plants. Consistent with the previous studies [25,27,34], melatonin application enhanced watermelon tolerance to chilling stress, and its action was closely correlated with the activated CBF-responsive pathway in this study (Figure 1). Furthermore, our results verified that the interaction between H_2_O_2_ and Ca^2+^ signals mediates melatonin-induced CBF-responsive pathway and subsequent cold tolerance.

### 4.1. RBOHD-Dependent H_2_O_2_ Is Required for Melatonin-Induced CBF-Responsive Pathway and Cold Tolerance

As an essential signaling molecule, RBOH-generated H_2_O_2_ plays a primary role in regulating plant response to various abiotic stimuli, such as cold stress [51,52,53]. For instance, cold acclimation enhanced *RBOH1* transcript levels and apoplastic H_2_O_2_ accumulation in tomato, while *RBOH1* silencing suppressed acclimation-induced cold tolerance [54]. *Arabidopsis* has 10 *RBOH* genes, *AtRBOHA* to *AtRBOHJ*. Among them, *AtRBOHD* encoded protein potentially regulates ROS-derived responses and plays a fundamental role in stress tolerance [51,55,56,57]. Plant *RBOH* genes are highly conserved [58]. Phylogenetic analysis and protein sequence alignment performed herein revealed that *Arabidopsis AtRBOHD* and watermelon *ClRBOHD* are homologous genes and their proteins share 79.3% similarity (Figure 6). Thus, we speculated that ClRBOHD might exhibit a similar function of producing H_2_O_2_ like AtRBOHD in *Arabidopsis*. In this study, chilling exposure induced H_2_O_2_ accumulation accompanied by *ClRBOHD* upregulation. Furthermore, exogenous application of H_2_O_2_ induced the expression of CBF-responsive pathway genes and chilling tolerance in watermelon. However, *AtRBOHD* knockout in *Arabidopsis* reduced the expression of CBF-responsive pathway genes and freezing tolerance. These results demonstrate that *RBOHD*-dependent H_2_O_2_ regulates the CBF-responsive pathway and cold tolerance in plants.

Melatonin plays primary functions in reducing ROS accumulation and alleviating stress-induced oxidative stress in plants [59]. Consistently, our results showed that melatonin alleviated chilling-induced H_2_O_2_ accumulation and lipid peroxidation after watermelon seedlings were exposed to 4 °C for 48 h (Figure 1). Many studies have shown that H_2_O_2_ plays an essential signaling role in melatonin-mediated regulation of various physiological processes, including stomatal closure, seed germination, lateral root formation, and response to environmental stimulus [19,29,60,61]. A recent report revealed that H_2_O_2_ mediates melatonin-induced cold tolerance in grafted watermelon plants [24]. In this study, melatonin induced H_2_O_2_ accumulation and *ClRBOHD* expression in early response (within 6 h) to chilling stress in watermelon (Figure 1A). Furthermore, exogenous application of H_2_O_2_ enhanced watermelon tolerance to chilling stress. However, DPI application in watermelon or *AtRBOHD* knockout in *Arabidopsis* inhibited H_2_O_2_ accumulation, suppressing the melatonin-induced expression of CBF pathway genes and subsequent cold stress tolerance (Figure 1, Figure 3 and Figure 8). These findings demonstrate that *RBOHD*-dependent H_2_O_2_ is required for melatonin-induced CBF-responsive pathway and the subsequent cold stress tolerance.

### 4.2. Ca^2+^ Mediates Melatonin-Induced CBF-Responsive Pathway and Cold Tolerance

Like H_2_O_2_, Ca^2+^, as an important second messenger, plays a critical role in regulation of responses to various abiotic stimulus in plants [62]. Low temperature induces a rapid and transient Ca^2+^ influx in plant cells by activating Ca^2+^ channels, such as cyclic nucleotide-gated ion channels (CNGCs) [8,63,64]. Various Ca^2+^ sensors decode the Ca^2+^ signal evoked by Ca^2+^ transient changes, subsequently regulating the expression of *CBFs* and *cold responsive* (*COR*) genes [8,65]. Several studies have confirmed that Ca^2+^ signaling is involved in melatonin-induced stomatal closure, seed germination, and salt tolerance in plants [19,29,66]. However, the relationship between Ca^2+^ and melatonin in response to cold stress remains unclear. In this study, melatonin promoted [Ca^2+^]_cyt_ accumulation under cold stress, accompanied by *ClCNGC2* upregulation (Figure 2). *Arabidopsis CNGC2*, a homologous gene of *ClCNGC2*, plays a critical role in contributing to Ca^2+^ entry into cytosol [48,49]. Here, exogenous CaCl_2_ induced the expression of CBF-responsive pathway genes and chilling tolerance in both watermelon and *Arabidopsis*. Meanwhile, blocking Ca^2+^ influx channels by LaCl_3_ counteracted melatonin-induced expression of CBF-responsive pathway genes and chilling tolerance in watermelon (Figure 3 and Figure 8). These results reveal that Ca^2+^ signaling is essential for melatonin-mediated CBF-responsive pathway and subsequent cold tolerance.

### 4.3. H_2_O_2_ and Ca^2+^ Function Together in a Self-Amplifying Feedback Loop in Melatonin-Induced CBF-Responsive Pathway and Cold Tolerance

**Figure 9:**
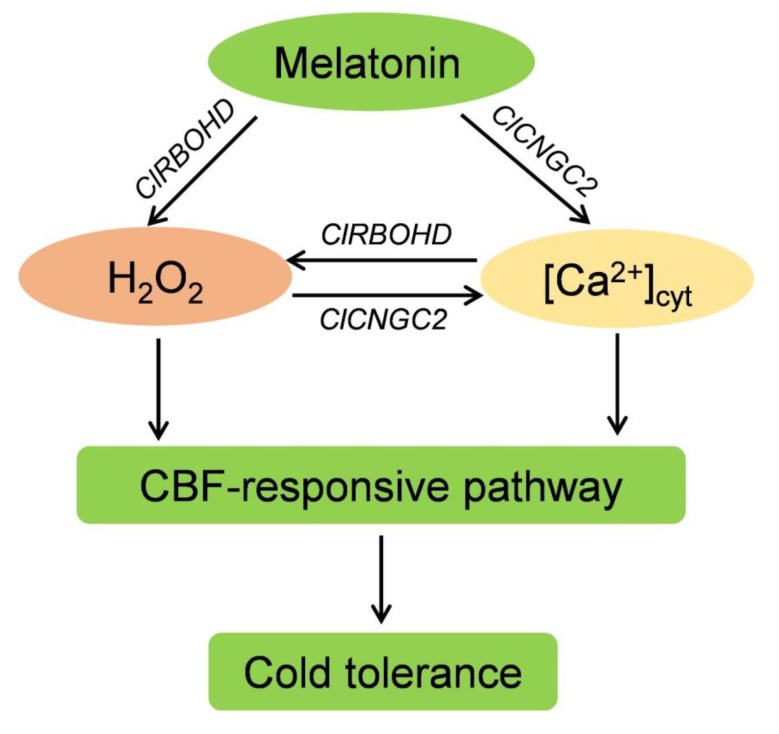
Schematic illustration of the proposed model of melatonin action on cold stress tolerance. During the early response to cold stress, melatonin increases the accumulation of H_2_O_2_ and cytoplasmic free Ca^2+^ ([Ca^2+^]_cyt_) by upregulating the expressions of *respiratory burst oxidase homolog* (*RBOH*) *D* and *cyclic nucleotide*-*gated channel* (*CNGC*) *2*, respectively. Increased H_2_O_2_ further promotes the accumulation of [Ca^2+^]_cyt_, which in turn elevates H_2_O_2_ accumulation, forming a reciprocal positive-regulatory loop that mediates melatonin-induced CBF pathway and subsequent cold tolerance.

As two crucial signal molecules, the interactions between H_2_O_2_ and Ca^2+^ signal have been well documented in various physiological actions, especially in defense against abiotic stressors [53,67]. For instance, stress-triggered Ca^2+^ signal can phosphorylate and activate RBOHD via Ca^2+^-dependent protein kinase to generate H_2_O_2_, which in turn elicits a Ca^2+^ signal through receptor-like kinases, such as GUARD CELL HYDROGEN PEROXIDE RESISTANT1, forming a self-propagating mutual activation loop between Ca^2+^ and H_2_O_2_ signals [62,67]. Consistent with the previous findings, we found that H_2_O_2_ and Ca^2+^ interact, forming a reciprocal positive-regulatory loop that mediates melatonin-induced CBF pathway and cold tolerance (Figure 9). The following evidence supports this conclusion: (1) H_2_O_2_ promoted [Ca^2+^]_cyt_ accumulation, while H_2_O_2_ deficiency in watermelon and *Arabidopsis* prevented melatonin-induced [Ca^2+^]_cyt_ accumulation in response to cold stress (Figure 2 and Figure 7); (2) CaCl_2_ stimulated H_2_O_2_ accumulation and upregulated *ClRBOHD* expression, while the blocking of Ca^2+^ influx by LaCl_3_ inhibited melatonin-induced increases in H_2_O_2_ accumulation and *ClRBOHD* transcripts (Figure 4); (3) LaCl_3_ counteracted H_2_O_2_-induced expression of CBF-responsive pathway genes and chilling tolerance in watermelon (Figure 3); (4) DPI treatment in watermelon or *AtRBOHD* knockout in *Arabidopsis* prevented Ca^2+^-induced expression of CBF pathway genes and cold tolerance (Figure 5 and Figure 8).

## 5. Conclusions

At present, the signaling mechanisms underlying melatonin-induced cold tolerance in watermelon are still elusive. This study reveals an intricate signaling cascade of melatonin-induced cold stress tolerance in watermelon. Melatonin induces H_2_O_2_ accumulation by upregulating *ClRBOHD* expression in early response to cold stress. Increased H_2_O_2_ induces *ClCNGC2* expression and [Ca^2+^]_cyt_ accumulation, which boosts H_2_O_2_ accumulation by triggering the *ClRBOHD* expression, forming a reciprocal positive-regulatory loop that induces the expression of CBF pathway genes and subsequent cold tolerance. This is the first study to investigate the interplay between H_2_O_2_ and Ca^2+^ signaling in melatonin-mediated cold tolerance to the best of our knowledge. However, other components of melatonin signaling in plant response to cold stress need to be further explored in the future.

## Data Availability

All datasets generated for this study are included in the article or Supplementary File.

## Supplementary Materials

The following are available online at https://www.mdpi.com/article/10.3390/antiox10091457/s1, Table S1: Gene-specific primers used for qRT-PCR analysis.

## Abbreviations

CBF, c-repeat binding factor; *COR*, *cold-responsive gene*; *ERD10*, *early responsive to dehydration 10*; *KIN1*, *cold induced gene 1*; H_2_O_2_, hydrogen peroxide; RBOH, respiratory burst oxidase homolog; DPI, diphenyleneiodonium; Ca^2+^, calcium ion; CNGC, cyclic nucleotide-gated ion channel; LaCl_3_, lanthanum chloride; Fv/Fm, maximum photosystem II quantum yield; REC, relative electrical conductivity; MDA, malondialdehyde; CK, control; MT, melatonin; CS, cold stress; WT, wild-type.
