# Supplementary material for: Positive Interaction between H2O2 and Ca2+ Mediates Melatonin-Induced CBF Pathway and Cold Tolerance in Watermelon (Citrullus lanatus L.)
Source: Antioxidants (Basel). 2021 Sep 14;10(9):1457. doi: 10.3390/antiox10091457 (PMC8471466; doi:10.3390/antiox10091457)
Supplement: Supplementary file 1 [file antioxidants-10-01457-s001.zip › antioxidants-1380685-supplementary.pdf]

Table S1 Gene-specific primers used for qRT-PCR analysis.

Gene	Accession number	Forward primer	Reverse primer
β-actin	*Cla97C02G026960*	CCATGTATGTTGCCATCCAG	GGATAGCATGGGGTAGAGCA
ClRBOHD	*Cla97C10G195960*	ACGAGGTAGCGGAAATGGACGA	GCATTGCTATCAACGCCGACCT
ClCBF1	*Cla97C01G005910*	ATATACAGAGGGCTGCGGCTCA	CCAAACAACGCCTCCTCATCCA
ClCOR47	*Cla97C08G152370*	TGGCGGAGTACGAGAACAAGAA	TCAGTAACGACGACCACCTCCT
ClERD10	*Cla97C08G152370*	AGGAGGTGGTCGTCGTTACTGA	TGCTATCGGATCGGGTGAGCTT
ClKIN1	*Cla97C01G004600*	AGGGTCAAGCACAGGAGAAAGC	CCAGTGGCATCCTTGACAGCAT
AtActin2	*AT3G18780*	GGTAACATTGTGCTCAGTGGTGG	AACGACCTTAATCTTCATGCTGC
AtCBF1	*AT4G25490*	GCATGTCTCAACTTCGCTGA	ATCGTCTCCTCCATGTCCAG
AtCOR47	*AT1G20440*	ACGGTCGCAACAGAGGAATCAC	GAGCGTCGTTGTCTCTTGAGGT
AtERD10	*AT5G15960*	AATGCCTTCCAAGCCGGTCAGA	CTGCCGCATCCGATACACTCTT
AtKIN1	*AT1G20450*	GCCACAGCAAGAAACCAGAGGA	TCTCCAGTGGTCTTGGCGTGAT
